# LACE index predicts age-specific unplanned readmissions and mortality after hospital discharge

**DOI:** 10.1007/s40520-020-01609-w

**Published:** 2020-06-05

**Authors:** Erica Heppleston, Christopher H. Fry, Kevin Kelly, Beth Shepherd, Ria Wright, Gareth Jones, Jonathan Robin, Paul Murray, David Fluck, Thang S. Han

**Affiliations:** 1 Quality Department, Ashford and St Peter’s Hospitals NHS Foundation Trust, Guildford Road, Chertsey, KT16 0PZ Surrey UK; 2 School of Physiology, Pharmacology and Neuroscience, University of Bristol, Bristol, BS8 1TD UK; 3 Digital Services, Department, Ashford and St Peter’s Hospitals NHS Foundation Trust, Guildford Road, Chertsey, KT16 0PZ Surrey UK; 4 Department of Medicine, Ashford and St Peter’s Hospitals NHS Foundation Trust, Guildford Road, Chertsey, KT16 0PZ Surrey UK; 5 Department of Respiratory, Ashford and St Peter’s Hospitals NHS Foundation Trust, Guildford Road, Chertsey, KT16 0PZ Surrey UK; 6 Department of Cardiology, Ashford and St Peter’s Hospitals NHS Foundation Trust, Guildford Road, Chertsey, KT16 0PZ Surrey UK; 7 Department of Endocrinology, Ashford and St Peter’s Hospitals NHS Foundation Trust, Guildford Road, Chertsey, KT16 0PZ Surrey UK; 8 Institute of Cardiovascular Research, Royal Holloway, University of London, Egham, TW20 0EX Surrey UK

**Keywords:** Prevention, Screening, Health economics, Quality of care, Patient safety

## Abstract

**Background:**

The LACE index scoring tool (*L*ength of stay, *A*cuity of admission, *C*o-morbidities and *E*mergency department visits) has been designed to predict hospital readmissions. We evaluated the ability of the LACE index to predict age-specific frequent admissions and mortality.

**Methods:**

Analysis of prospectively collected data of alive-discharge episodes between 01/04/2017 and 31/03/2019 in an NHS hospital. Data on 14,878 men and 17,392 women of mean age 64.0 years, SD = 20.5, range 18.0–106.7 years were analysed. The association of the LACE index with frequency of all-cause readmissions within 28 days of discharge and over a 2-year period, and with all-cause mortality within 30 days or within 6 months after discharge from hospital were evaluated.

**Results:**

Within LACE index scores of 0–4, 5–9 or ≥ 10, the proportions of readmission ≥ 2 times within 28 days of discharge were 0.1, 1.3 and 9.2% (χ^2^ = 3070, *p* < 0.001) and over a 2-year period were 1.7, 4.8 and 19.1% (χ^2^ = 3364, *p* < 0.001). Compared with a LACE index score of 0–4, a score ≥ 10 increased the risk (adjusted for age, sex and frequency of admissions) of death within 6 months of discharge by 6.8-fold (5.1–9.0, *p* < 0.001) among all ages, and most strongly in youngest individuals (18.0–49.9 years): adjusted odds ratio = 16.1 (5.7–45.8, *p* < 0.001). For those aged 50–59.9, 60–69.9, 70–79.9 and ≥ 80 years, odds ratios reduced progressively to 9.6, 7.7, 5.1 and 2.3, respectively. Similar patterns were observed for the association of LACE index with mortality within 30 days of hospital discharge.

**Conclusions:**

The LACE index predicts short-term and long-term frequent admissions and short-term and medium-term mortality, most pronounced among younger individuals, after hospital discharge.

## Introduction

Healthcare services are continually overstretched due to increasing demand, primarily from an expanding ageing population living with multiple chronic conditions and disabilities [1–3]. Many such individuals have frequent early hospital readmissions [4] and prolonged length of stay in hospital [5, 6], imposing a high pressure on healthcare systems [7, 8]. Information on the number of individuals with high risk of readmission and mortality would allow healthcare teams to formulate effective clinical plans and resources. The LACE index scoring tool, based on *L*ength of stay, *A*cuity of admission, *C*o-morbidities and *E*mergency department visits, has been designed to predict early hospital readmissions and death [9] and has been implemented widely across hospitals in the UK and in many other countries [10–14].

The LACE index represents a cluster of features that indicate the health status of an individual; the higher the index score, the poorer is their health and a greater risk of death. The role of the LACE index in relation to admissions and mortality has been explored, but studies tend to focus primarily on older individuals and short periods after discharge from hospital (up to about one month) before readmission [14, 15], or death [10–14]. Among the overall population in England and Wales, the proportions of younger adults aged 18–29, 30–39, 40–49 and 50–59 years are 16.2, 13.3, 14.6 and 12.1%, while the respective proportions of older adults aged 60–69, 70–79 and over 80 years are lower, are 10.8, 7.1 and 4.6% [16]. However, there is a paucity of data on the ability of the LACE index to predict age-specific mortality occurring after discharge and at times greater than one month after discharge, and frequent readmissions over a prolonged period [17]. In this study, we quantify the ability of the LACE index to predict, in adults aged between 18 and 107 years, the risk of all-cause frequent unplanned readmissions (within 28 days of discharge) and multiple readmissions over a period of two years, and also to predict the risk of all-cause mortality within 30 days or within six months of discharge from hospital.

## Methods

### Study design, participants and setting

Data of consecutive alive-discharge episodes over two years between 1st April 2017 and 31st March 2019 in a single National Health Service hospital were prospectively collected. The data comprised clinical characteristics and care quality including age, sex, primary diagnosis on admission, the length of stay in hospital, nature of the admission, co-morbidities and number of previous emergency department visits.

### Measurement

Co-morbidities were coded according to ICD-10 for calculation of the Charlson co-morbidity index [18, 19]. Information on the frequency of unplanned admissions and readmissions within 28 days and over a two-year period, and mortality within 30 days and up to six months after hospital discharge was recorded. Cancer and obstetrics spells were excluded in line with the NHS data collection for general hospital admissions [20].

The LACE index was computed (https://www.mdcalc.com/lace-index-readmission) from length of stay (score range 0–7), acuity of admission (score 0 or 3), co-morbidity (score range 0–5), emergency department visits (score range 0 or 4) with the scale ranging from 0 to 19 and the likelihood of outcome risk (mortality) is raised with increasing score [9].

### Categorisation of variables

LACE indices were grouped into low (score = 0–4), moderate (score = 5–9) and high (score ≥ 10) risk [15, 21, 22]. Age was categorised by decades from 50 years old: 50–59.9, 60–69.9, 70–79.9 and ≥ 80 years. All those between 18 and 49.9 years were grouped together due to low mortality rates, while those between 80 and 107 years were combined together due to small numbers – only 2461 (7.6%) patients were older than 90 years. Readmissions within 28 days of discharge or over a period of two financial years were categorised into three groups: No readmission, readmitted once, and readmitted ≥ 2 times.

### Statistical analysis

Chi-square tests were used to assess the relationship between the proportions of all-cause readmissions and rates of all-cause mortality in relation to the LACE index. Receiver operating characteristic (ROC) curves were constructed to determine the area under the curve (AUC) for the LACE index as a predictor of outcomes (mortality or frequent admissions). Cox regression survival analysis and Kaplan–Meier survival curves were constructed to examine the risk of mortality after discharge. Logistic regression was conducted using categories of LACE index scores; 0–4 (reference group), 5–9 and ≥ 10 as the predictor variable of frequent readmissions (≥ 2 times within 28 days of discharge or ≥ 2 times over a 2-year period), or mortality within 30 days or within six months of hospital discharge (dependent variables). For analysis of frequent admissions, data were adjusted for age and sex. For analysis of mortality, data were presented in three models; model 1: unadjusted, model 2: adjusted for age and sex, and model 3: adjusted for age, sex and frequency of admission in all ages first, followed by age-specific analysis. Odds ratios (OR) are given with 95% confidence intervals (CI). Analyses were performed using IBM SPSS Statistics, v23.0 (IBM Corp., Armonk, NY).

## Results

### Subject characteristics

Data for a total of 32,270 patients (14,878 men) and (17,392 women) aged 18–106.7 yr (mean = 64.0 years, SD = 20.5) were analysed. There were 29.3% of patients with a LACE index score of ≥ 10. A total of 11.6% were readmitted within 28 days (8.1% readmitted once and 3.3% readmitted ≥ 2 times), and 21.6% were readmitted over a 2-year period (13.5% readmitted once and 8.1% readmitted ≥ 2 times). There were 834 (2.6%) and 2192 (6.8%) patients died within 30 days (mean age of death 81.5 years, SD 12.0) and six months of discharge (mean age of death 81.2 years, SD 12.1), respectively (Table 1).

**Table 1:** Subject characteristics of 14,878 men (mean age = 63.9 years, SD = 19.3, range 18.0–104.1) and 17,392 women (mean age = 64.1 years, SD = 21.6, range 18.0–106.7)

	*n*	%
*Age distribution*		
18–49.9 years	8403	26.0
50–59.9 years	4304	13.3
60–69.9 years	4739	14.7
70–79.9 years	6068	18.8
≥ 80 years	8756	27.1
*LACE index categories*		
LACE < 4	9330	28.9
LACE 4–9	13,500	41.8
LACE ≥ 10	9440	29.3
*Number of readmissions within 28 days of discharge*		
None	28,548	88.5
Once	2666	8.3
≥ 2 times	1056	3.3
*Total number of readmissions over two years*		
None	25,295	78.4
Once	4360	13.5
≥ 2 times	2615	8.1
*Mortality status*		
Death within 30 days of admission	834	2.6
Death within six months of admission	2192*	6.8

^*^This group includes those who died within 30 days of admission

The proportions for those who were readmitted ≥ 2 times within 28 days of discharge were 1.0% (18–49.9 years), 1.5% (50–59.9 years), 2.4% (60–69.9 years), 3.3% (70–79.9 years) and 6.7% (≥ 80 years) (χ^2^ = 1087, p < 0.001), and for those who were readmitted ≥ 2 times over a 2-yr period were 3.3% (18–49.9 years), 5.1% (50–59.9 years), 6.7% (60–69.9 years), 8.7% (70–79.9 years) and 14.4% (≥ 80 years) (χ^2^ = 1335, *p* < 0.001).

The proportions of patients with a LACE index score ≥ 10 also rose steeply with age: 1.7% (18–49.9 years), 5.6% (50–59.9 years), 18.0% (60–69.9 years), 39.1% (70–79.9 years) and 66.6% (80–89.9 years) (χ^2^ = 15,804, *p* < 0.001) and with frequency of readmission within 28 days of discharge: 24.5% (no readmission), 59.3% (one readmission) and 82.6% (≥ 2 readmissions) (χ^2^ = 3070, *p* < 0.001), and with frequency of readmission over the 2-year period of study: 22.2% (no readmission), 46.0% (one readmission) and 69.1% (≥ 2 readmissions) (χ^2^ = 3364, *p* < 0.001).

ROC analysis to generate AUC values showed that the LACE index as a predictor of mortality within six months of hospital discharge was 80.5% (95%CI = 79.7–81.3) and frequent readmissions was 84.0%, (95%CI = 83.0–85.1).

### LACE index as predictor of all-cause unplanned readmissions

**Fig. 1:**
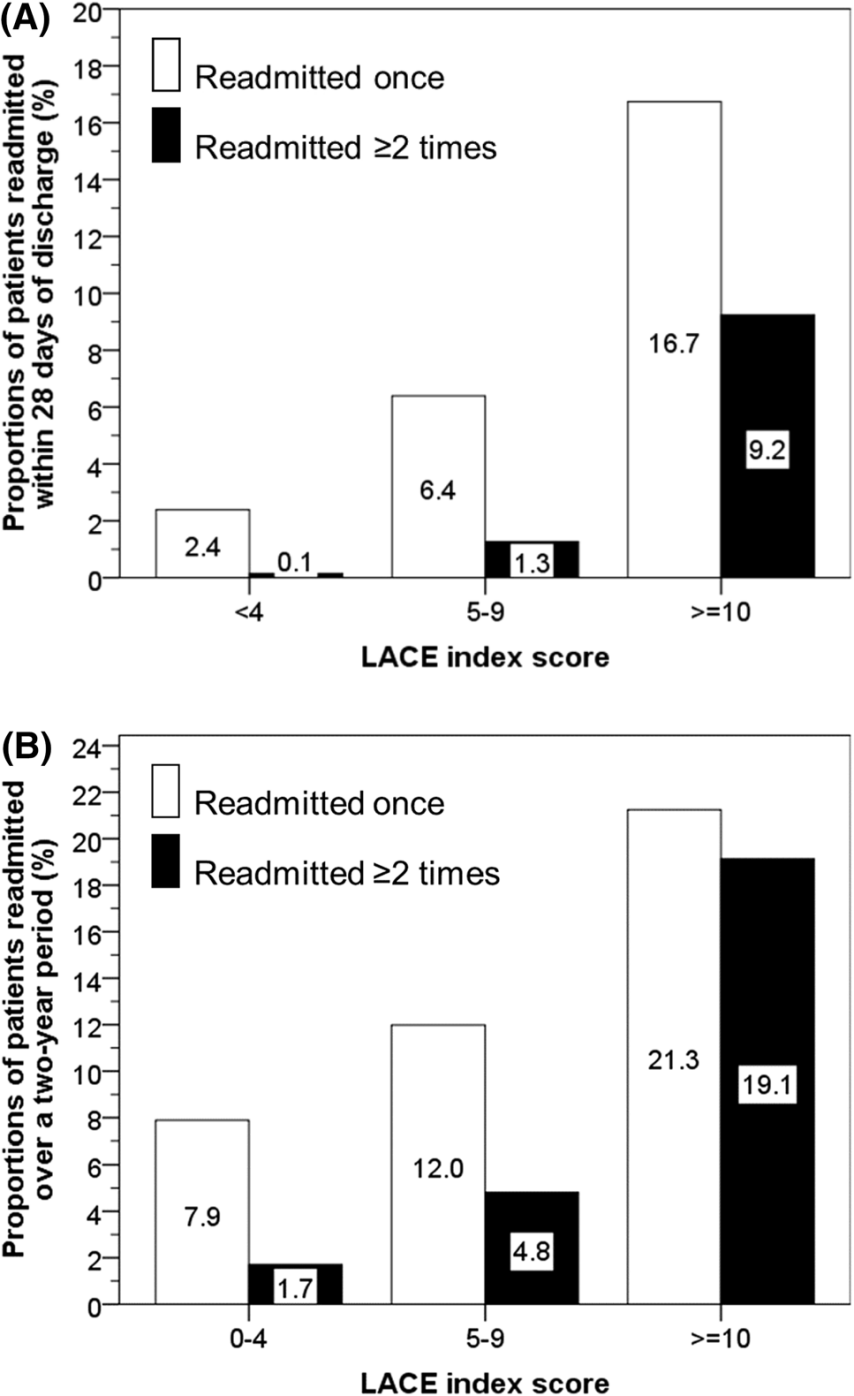
Proportions of patients readmitted within 28 days of discharge; group differences: χ^2^ = 3070, *p* < 0.001 (**a**) or readmitted over a two year period; group differences: χ^2^ = 3364, p < 0.001 (**b**)

Within LACE index score groups of 0–4, 5–9 or ≥ 10, the proportions of readmission ≥ 2 times within 28 days of discharge were 0.1, 1.3 and 9.2% (χ^2^ = 3070, *p* < 0.001) and over a 2-year period were 1.7, 4.8 and 19.1% (χ^2^ = 3364, *p* < 0.001) (Fig. 1). Compared with individuals with a LACE index score of 0–4 (reference group), those with a score of 5–9 had increased risk (adjusted for age and sex) of frequent admissions (≥ 2 times) within 28 days after discharge: OR = 10.4 (95% CI = 5.9–18.5, *p* < 0.001) and over a 2-yr period: OR = 3.1 (95% CI = 2.6–3.7, *p* < 0.001). This further increased in those with a score ≥ 10, frequent admissions within 28 days after discharge: OR = 94.2 (95% CI = 53.0–167.4, *p* < 0.001) and over a 2-yr period OR = 15.3 (95% CI = 12.6–18.6, *p* < 0.001).

### LACE index as predictor of all-cause mortality

The proportions of patients who died within 30 days of discharge rose from 0.1% in the lowest LACE index group (0–4) to 1.4% in intermediate group (5–9) and up to 6.8% in the highest group (≥ 10) (χ^2^ = 957, *p* < 0.001). The corresponding figures for those who died within six months of discharge were 0.7, 3.9 and 17.0% (χ^2^ = 2275, *p* < 0.001). Compared with a LACE index score = 0–4, a score ≥ 10 increased the risk (adjusted for age, sex and frequency of admissions) of death within three months by 13.5-fold (95%CI = 7.4–24.6, *p* < 0.001) or within six months of discharge by 6.8-fold (5.1–9.0, *p* < 0.001).

**Fig. 2:**
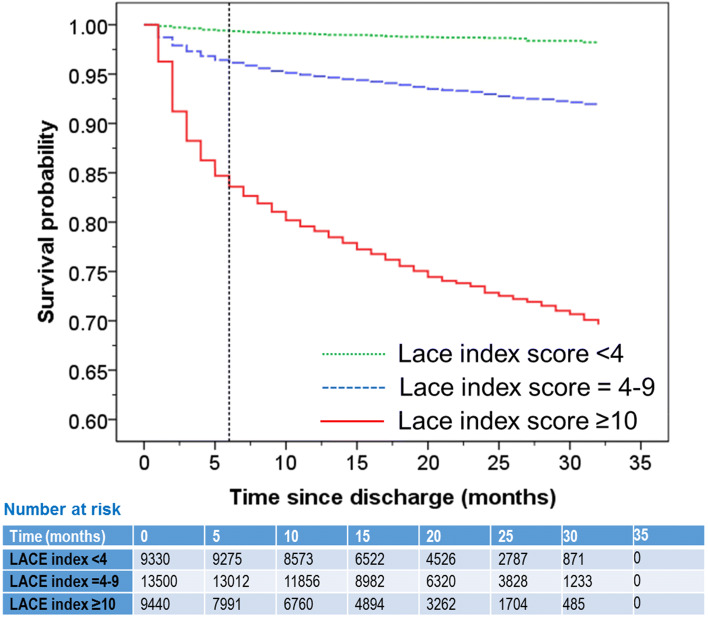
Kaplan–Meier survival plot in patients with a LACE index < 4 (dotted green line), 4–9 (dashed blue line) and ≥ 10 (solid red line). The vertical dotted black line shows the 6-month time for one of the mortality estimates. The table beneath the figure shows the number of at-risk patients at various time points for the three LACE index cohorts

Compared to those with a LACE index < 4 (mean for survival time from discharge = 32.1 months, 95%CI = 32.0–32.2), those with a LACE index of 4–9 or ≥ 10 had a significantly shorter survival with mean for survival time from discharge = 30.6 (95%CI = 30.5–30.8) and 29.5 (95%CI = 25.0–25.5) months respectively, log rank (Mantel-Cox) test: χ^2^ = 3382, *p* < 0.001. The hazard ratio, adjusted for age, sex and frequency of readmissions, was for a LACE index 4–9 = 2.58 (95% CI = 2.11–3.14, *p* < 0.001) and for a LACE index ≥ 10 = 6.38 (95% CI = 5.21–7.18, *p* < 0.001) (Fig. 2).

A LACE index predicted mortality most strongly in younger individuals (18–49.9 years): a score ≥ 10 was associated with greater risk of death within 30 days after discharge: OR = 30.5 (95%CI = 4.6–202.9, *p* < 0.001), and death within six months after discharge: OR = 16.1 (5.7–45.8, *p* < 0.001). For those aged 50–59.9, 60–69.9, 70–79.9 and ≥ 80 years, ORs reduced to 20.5, 7.5, 15.6 and 5.3 for death within 30 days and to 9.6, 7.7, 5.1 and 2.3 for death within six months after hospital discharge, respectively (Table 2).

**Table 2:** Logistic regression for risk of all-cause mortality within 30 days or within six months of discharge in all patients and by 10 yr age bands

	*n*	Mortality within 30 days of discharge							Mortality within six months of discharge						
		Mortality rates (%)	LACE score = 4–9 (*n* = 13,500)*			LACE score ≥ 10 (*n* = 9440)*			Mortality rates (%)	LACE score = 4–9 (*n* = 13,500)*			LACE score ≥ 10 (*n* = 9440)*		
			OR	95% CI	*p*	OR	95% CI	*p*		OR	95% CI	*p*	OR	95% CI	*p*
Model 1: Unadjusted															
All patients	32,270	2.6	10.7	6.0–19.2	< 0.001	56.3	31.8–99.7	< 0.001	6.8	6.1	4.7–7.9	< 0.001	30.6	23.7–39.4	< 0.001
Age bands															
18–49.9 yr	8403	0.2	10.5	2.3–48.0	0.002	59.5	9.9–358.6	< 0.001	0.5	6.3	3.0–13.5	< 0.001	36.5	13.9–96.0	< 0.001
50–59.9 yr	4304	0.7	7.4	1.7–31.9	0.007	30.7	6.5–145.5	< 0.001	2.0	5.4	2.7–10.9	< 0.001	15.0	6.6–34.1	< 0.001
60–69.9 yr	4739	1.8	4.0	1.4–11.1	0.008	11.7	4.2–33.0	< 0.001	4.8	2.3	1.3–4.0	0.003	11.0	6.3–18.0	< 0.001
70–79.9 yr	6068	2.9	5.4	1.3–22.5	0.019	21.2	5.2–85.7	< 0.001	7.7	1.8	1.1–3.1	0.025	7.2	4.3–12.0	< 0.001
≥ 80 yr	8756	6.0	2.5	0.6–10.3	0.202	8.4	2.1–33.8	0.003	15.6	1.3	0.7–2.3	0.366	3.4	1.9–6.0	< 0.001
Model 2: Adjusted for age and sex															
All patients	32,270	2.6	5.7	3.1–10.3	< 0.001	18.7	10.3–34.0	< 0.001	6.8	3.0	2.3–4.0	< 0.001	9.3	7.0–12.3	< 0.001
Age bands															
18–49.9 yr	8403	0.2	10.1	2.2–46.4	0.003	54.3	8.9–330.8	< 0.001	0.5	5.9	2.7–12.5	< 0.001	30.4	11.5–80.6	< 0.001
50–59.9 yr	4304	0.7	7.5	1.8–32.4	0.007	31.4	6.6–149.9	< 0.001	2.0	5.4	2.7–10.9	< 0.001	15.1	6.6–34.3	< 0.001
60–69.9 yr	4739	1.8	3.9	1.4–10.9	0.009	11.3	4.0–31.9	< 0.001	4.8	2.3	1.3–4.0	0.003	10.7	6.2–18.5	< 0.001
70–79.9 yr	6068	2.9	5.5	1.3–22.7	0.019	21.4	5.3–86.6	< 0.001	7.7	1.9	1.1–3.1	0.022	7.3	4.4–12.1	< 0.001
≥ 80 yr	8756	6.0	2.4	0.6–9.8	0.230	7.6	1.9–30.7	0.005	15.6	1.2	0.7–2.2	0.366	3.1	1.8–5.5	< 0.001
Model 3: Adjusted for age, sex and frequency of readmissions															
All patients	32,270	2.6	5.3	2.9–9.6	< 0.001	13.5	7.4–24.6	< 0.001	6.8	2.8	2.2–3.7	< 0.001	6.8	5.1–9.0	< 0.001
Age bands															
18–49.9 yr	8403	0.2	8.1	1.7–37.7	0.008	30.5	4.6–202.9	< 0.001	0.5	4.6	2.1–10.0	< 0.001	16.1	5.7–45.8	< 0.001
50–59.9 yr	4304	0.7	6.4	1.5–27.9	0.013	20.5	4.1–103.1	< 0.001	2.0	4.5	2.2–9.1	< 0.001	9.6	4.1–22.7	< 0.001
60–69.9 yr	4739	1.8	3.4	1.2–9.5	0.020	7.5	2.6–21.7	< 0.001	4.8	2.1	1.2–3.6	0.010	7.7	4.4–13.5	< 0.001
70–79.9 yr	6068	2.9	5.1	1.2–21.2	0.024	15.6	3.8–63.6	< 0.001	7.7	1.7	1.0–2.9	0.043	5.1	3.1–8.6	< 0.001
≥ 80 yr	8756	6.0	2.2	0.5–8.9	0.286	5.3	1.3–21.3	0.020	15.6	1.2	0.6–2.1	0.626	2.3	1.3–4.1	0.004

*OR *odds ratio; 95%CI = 95% confidence interval

*Reference group: LACE index score 0–4 (*n* = 9330)

## Discussion

This study, over a period of 2 years, found a high LACE index was related to all-cause frequent readmissions within 28 days, as well as over a two-year period, after hospital discharge. The same relation was observed for all-cause mortality within 30 days or six months post-hospital discharge. The risk of mortality was most pronounced among younger individuals; patients aged 18–49.9 years with a LACE index score ≥ 10 had a 30.5-fold increased risk of death within 30 days and a 16.1-fold increased risk of death within 6 months of discharge.

Evidence from this study supports the use of a LACE index as a valuable tool for identifying individuals at risk. The proportions of patients with a high LACE index score (≥ 10) are relatively high, but have been reported to range between 16.0–48.5% [13, 21, 22], compared with 29.3% in this study. However we observed that these values vary with age; the proportion of individuals with LACE index scores ≥ 10 was only 1.7% among the youngest group (18–49.9 years) and more than doubled with each following decade of age to a peak level of 69.1% among those aged ≥ 80 years. It is therefore important to take age into account when the rates of patients with high LACE index are analysed or reported.

Our findings of the overall rate (11.6%) of readmissions within 28 days of discharge was similar to that (12.6%) reported by Gruneir et al. [21] and by Lim et al. (11.6%) [23], but lower than the figure (18.4%) reported by Tan et al. [22], probably due to age differences between study populations. The observation of increased risk of frequent readmission among those with LACE index scores ≥ 10 was consistent with previous studies [21, 22]. In this study, we have also found that almost a fifth of patients with LACE index score ≥ 10 to be at risk of multiple readmissions (≥ 2 times) in the long-term (two-year period). These findings provide valuable information to healthcare teams to identify those at long-term risk of readmissions to support preventative and early interventional measures to those who are most vulnerable. This will improve patient care and reduce pressure and costs to healthcare services. Efforts have been made to reduce hospital readmissions such as the Hospital Readmissions Reduction Program (HRRP) in the US but results have been mixed due to increased mortality [24]. It is therefore important to address the balance of benefit and risk of readmissions reduction to avoid missing high risk patients who remain reliant on hospital readmission for necessary treatment.

The mortality rates observed in our study were also comparable to those recently reported for 30 days [25] and 6 months of discharge [26, 27]. There was a clear increasing trend in the risk of mortality from higher LACE index scores in the youngest age group. This trend continued to persist with older age groups but was progressively less pronounced. These increased risks were adjusted for age, sex and frequency of admissions. As far as we are aware, this is the first study to demonstrate an age-specific relationship between the LACE index and mortality and was achieved over a wide range of age (18–107 years). Lowering the cut-off level of a LACE index score for younger individuals may be necessary to identify more patients at high risk of mortality after hospital discharge.

This study also demonstrated that the LACE index has predictive validity for short-term (30-days) and medium-term (6-months) mortality, with clear stepwise increments in mortality. This suggests further research is required to gain greater insights into those younger individuals who have high LACE index scores, to lower their risk of death after discharge from hospital.

The strengths of this study lie in its large number of consecutive patients, which enable us to estimate the risk of mortality by decades of age, ranging from 18 to 107 years. Appropriate adjustments were made including age, sex and frequency of admission. Further adjustment for primary diagnosis on admission did not change these associations. Our hospital is typical of a General District Hospital in the UK. Our previous studies examining other health outcomes, using data from three other hospitals within the same county, showed very similar characteristics and indeed with the rest of the UK [28, 29]. Any bias is therefore likely to be minimal in our study. The present study did not collect information on socioeconomic status, employment or provenience (urban or rural) that could have some bearing on the outcomes. We employed the validated LACE index as a prognostic tool to predict outcome measures while cut-off points of 0–4, 5–9 and ≥ 10 were based on previous studies [15, 21]. These cut-off points are arbitrary therefore raising the score above 10 for the “high-risk” group would identify higher rates of mortality. Conversely lowering the cut-off score below 4 for the “low-risk” group would reduce rates of mortality, thus exaggerating the predictive ability (ORs) of mortality by the LACE index. Further studies to identify age-specific cut-offs for the LACE index as an indicator of adverse outcomes (such as mortality) are required.

In conclusion, the LACE index predicts short-term and long-term frequent admissions and short-term and medium-term mortality, most pronounced among younger individuals, after hospital discharge. Raising awareness of younger individuals with a high LACE score is recommended.
